# Adjuvant chemotherapy combined with immunotherapy in patients with cholangiocarcinoma after radical resection

**DOI:** 10.1002/cam4.6738

**Published:** 2023-12-07

**Authors:** Xiao‐hui Li, En‐liang Zhou, Chong‐yu Zhao, Bo‐kang Cui, Xiao‐yuan Dong, Hang Du, Xiao‐jun Lin

**Affiliations:** ^1^ Department of Pancreatobiliary Surgery, State Key Laboratory of Oncology in South China, Guangdong Provincial Clinical Research Center for Cancer Sun Yat‐sen University Cancer Center Guangzhou China; ^2^ Department of Hepatobiliary Surgery The Second Affiliated Hospital of Army Medical University Chongqing China; ^3^ Department of Gynecology Guangdong Hydropower Hospital Guangzhou China; ^4^ Reproductive and Genetic Medicine Center Dalian Women and Children's Medical Group Dalian China

**Keywords:** chemotherapy, cholangiocarcinoma, immunotherapy, survival

## Abstract

**Background:**

The malignancy of cholangiocarcinoma is highly pronounced, and it exhibits a propensity for recurrence and metastasis even in the presence of standard chemotherapy. The efficacy of adjuvant chemotherapy combined with immunotherapy in patients with resected cholangiocarcinoma needs to be substantiated.

**Methods:**

Data from 101 patients with cholangiocarcinoma treated at the Sun Yat‐sen University Cancer Center between 2015 and 2020 were studied.

**Results:**

After propensity score matching, there were no significant differences in baseline characteristics between patients in the combined adjuvant chemotherapy and immunotherapy group (AC + IM group) and the adjuvant chemotherapy alone group (AC group) (all *p* > 0.05). The AC + IM group demonstrated a statistically significant improvement in relapse‐free survival (RFS) compared to the AC group (*p* = 0.032). Likewise, the AC + IM group exhibited a significantly superior overall survival (OS) outcome when compared to the AC group (*p* = 0.044). Multivariate Cox analysis unveiled perineural invasion (*p* = 0.041), lymph node metastasis (*p* = 0.006), and postoperative immunotherapy (*p* = 0.008) as independent prognostic factors exerting a significant impact on the OS of patients. In the cohort of patients with perineural invasion, the AC + IM group exhibited significantly improved OS compared to the AC group (*p* = 0.0077). Similarly, within the subset of patients with lymph node metastasis, the AC + IM group exhibited a significantly superior OS outcome when compared to the AC group (*p* = 0.023).

**Conclusion:**

Combining postoperative adjuvant chemotherapy with immunotherapy extends the RFS and OS of patients with cholangiocarcinoma following radical resection.

## 1 INTRODUCTION

Cholangiocarcinoma is a highly malignant tumor that arises from the epithelium of the bile duct. It can be classified into intrahepatic, hilar, distal, and gallbladder cholangiocarcinoma. The prognosis for cholangiocarcinoma is very poor, with surgery being the only possible curative treatment. Unfortunately, even with curative surgery, the recurrence rate of cholangiocarcinoma reaches 67% within 1 year, and the 5‐year overall survival (OS) rate is only 25%–40%.^1^, ^2^, ^3^ Although fluorouracil‐based chemotherapy serves as the standard therapeutic modality for cholangiocarcinoma patients, its efficacy in treating tumors is remarkably limited, resulting in a median OS of approximately 12 months.^4^

Postoperative adjuvant therapy holds promise in mitigating the risk of recurrence and metastasis. In the BILCAP study,^5^ the inclusion of capecitabine as adjuvant therapy demonstrated a significant survival advantage of 14.7 months compared to the surgery alone group in the intention‐to‐treat analysis (*p* = 0.097). Supporting these findings, Kamarajah et al.^6^ reported that postoperative adjuvant chemotherapy correlated with improved survival outcomes (median OS: 28.2 months). Moreover, Schweitzer et al.^7^ demonstrated a significant improvement in OS within the adjuvant chemotherapy group compared to the surgery alone group (33.5 months vs. 18.0 months, *p* = 0.002). Consistently, the American Society of Clinical Oncology guidelines strongly advocate for adjuvant chemotherapy comprising a 6‐month course of capecitabine in patients who have undergone resection for cholangiocarcinoma.^8^

In recent times, significant advancements have been achieved in the realm of immune checkpoint blockade (ICB) for the management of diverse solid tumors, encompassing melanoma, renal cancer, and hepatocellular carcinoma.^9^ Additionally, multiple clinical trials have evaluated the effectiveness of ICB in the treatment of advanced cholangiocarcinoma. Among these trials, KEYNOTE‐158^10^ is a phase II clinical study evaluating the effectiveness of pembrolizumab in patients with advanced cholangiocarcinoma who have experienced first‐line treatment failure. The study included 104 patients with cholangiocarcinoma, revealing significant findings: six patients (5.8%) showed a partial response (PR), and 17 patients (16.3%) had stable disease. A separate phase II clinical trial^11^ investigating the effectiveness of nivolumab in advanced cholangiocarcinoma has shown promising clinical outcomes, with a median OS of 14.24 months. Moreover, two studies have indicated that combining ICB with chemotherapy is an effective and well‐tolerated treatment strategy for patients with advanced cholangiocarcinoma.^12^, ^13^ Hence, this study aimed to analysis the value of combining immunotherapy with chemotherapy in cholangiocarcinoma patients undergoing radical resection.

## 2 DATA AND METHODS

### 2.1 Data sources

**FIGURE 1:**
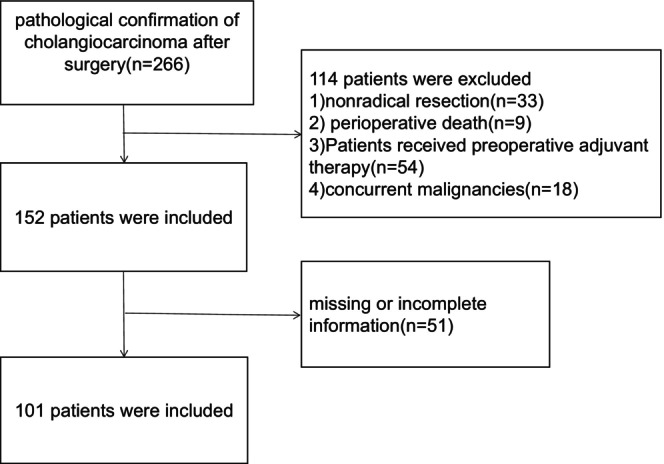
Flowchart of the patient enrolling process.

We conducted a retrospective analysis of data from patients with cholangiocarcinoma who underwent surgery at Sun Yat‐sen University Cancer Center between January 2015 and January 2020. Inclusion criteria were as follows: (1) pathologically confirmed cases of cholangiocarcinoma who underwent surgical resection; (2) those who received postoperative adjuvant therapy (including adjuvant chemotherapy or immunotherapy). Exclusion criteria were as follows: (1) palliative surgery; (2) perioperative death; (3) preoperative neoadjuvant therapy; (4) complicated with other malignant tumors; and (5) incomplete clinical characteristics or follow‐up data. Figure 1 depicts a comprehensive flow diagram illustrating the process of patient selection in this study.

### 2.2 Postoperative adjuvant chemotherapy and immunotherapy

Prior to receiving postoperative adjuvant therapy, a comprehensive evaluation of the physical condition was conducted for all cholangiocarcinoma patients who underwent radical surgical resection. They were categorized into two groups based on the postoperative treatment regimen. The initial group underwent postoperative adjuvant chemotherapy in conjunction with immunotherapy (AC + IM), whereas the second group solely underwent postoperative adjuvant chemotherapy (AC). The postoperative adjuvant chemotherapy regimens for cholangiocarcinoma comprised gemcitabine + capecitabine and capecitabine monotherapy. As for postoperative immunotherapy, PD‐1 and PD‐L1 immune checkpoint inhibitors were employed. The administration protocols and dosages of these drugs were tailored by clinical oncology specialists on an individual basis, taking into account tumor dynamics and patient‐specific drug responses.

### 2.3 Follow‐up and outcome indicators

Patients returned to the hospital's outpatient clinic within the initial month following surgery and subsequently every 3 months. In the event of tumor recurrence, tailored interventions were implemented based on the specific circumstances. Standard review components encompassed blood tests, digestive tract tumor markers, and upper abdominal computed tomography or magnetic resonance imaging scans (both plain and enhanced). OS and relapse‐free survival (RFS) were employed as the principal outcome measures to evaluate the prognosis of patients undergoing surgical intervention for cholangiocarcinoma.

### 2.4 Statistical analysis

Analyses were conducted using SPSS Statistics and R software (version 4.1.2). For continuous variables, Student's *t*‐test was employed, while categorical variables were assessed using parametric and McNemar tests as nonparametric alternatives. Fisher's exact test was utilized for within‐group comparisons. The COX proportional hazards regression model was employed to determine the independent prognostic risk factors. Propensity score matching (PSM) was carried out using IBM SPSS 25.0 software. Statistical significance was defined as a *p*‐value less than 0.05.

## 3 RESULTS

### 3.1 Baseline data

Table 1 presents the clinicopathological features. After PSM, 64 patients (32 each in the AC + IM and AC groups) were selected. Table 2 presents the clinical characteristics of patients within each group, as observed in the matched cohorts.

**TABLE 1:** Baseline characteristics of two groups.

Variables	AC + I (*n* = 35)	AC (*n* = 66)	*X* ^2^	*p*
Gender				
Male	20	36		
Female	15	30	0.062	0.803
Age (years)				
≤55	23	40		
>55	12	26	0.254	0.614
Tumor location^a^				
iCCA	20	44		
pCCA	14	16		
dCCA	1	6	3.522	0.172
Tumor differentiation				
Low	3	8		
Moderate	29	55		
High	3	3	0.889	0.641
Microvascular invasion				
Absence	23	42		
Presence	12	24	0.043	0.836
Perineural invasion				
Absence	18	30		
Presence	17	36	0.327	0.567
T stage				
1	14	30		
2	16	23		
3	3	10		
4	2	3	1.688	0.640
Lymph node metastasis				
Absence	22	48		
Presence	13	18	0.539	0.463
Preoperative CEA (ng/mL)				
≤5.00	26	42		
>5.00	9	24	1.179	0.278
Preoperative Ca19‐9 (U/mL)				
≤37.00	12	17		
>37.00	23	49	0.813	0.367
Preoperative TBIL (μmol/L)				
≤17.1	21	37		
>17.1	14	29	0.145	0.703
HbsAg				
Negative	25	47		
Positive	10	19	0.001	0.982
Chemotherapy regimens^b^				
GX	14	27		
X	21	39	0.008	0.929

^a^ Tumor location: cholangiocarcinoma (CCA) is best classified according to the primary, anatomic subtype as intrahepatic CCA (iCCA), perihilar CCA (pCCA), and distal CCA (dCCA).

^b^ Chemotherapy regimens: gemcitabine + capecitabine (GX); capecitabine monotherapy (X).

**TABLE 2:** Baseline characteristics of two groups after propensity score matching.

Variables	AC + I (*n* = 32)	AC (*n* = 32)	*X* ^2^	*p*
Gender				
Male	19	20		
Female	13	12	0.066	0.798
Age (years)				
≤55	23	23		
>55	9	9	0	1.000
Tumor location^a^				
iCCA	17	14		
pCCA	14	13		
dCCA	1	5	2.994	0.224
Tumor differentiation				
Low	3	3		
Moderate	26	27		
High	3	2	0.219	0.896
Microvascular invasion				
Absence	20	20		
Presence	12	12	0	1.000
Perineural invasion				
Absence	17	10		
Presence	15	22	0.567	0.451
T stage				
1	11	8		
2	16	21		
3	3	1		
4	2	2	2.149	0.542
Lymph node metastasis				
Absence	19	18		
Presence	13	14	0.026	0.932
Preoperative CEA (ng/mL)				
≤5.00	24	19		
>5.00	8	13	1.772	0.183
Preoperative Ca19‐9 (U/mL)				
≤37.00	12	8		
>37.00	20	24	1.164	0.281
Preoperative TBIL (μmol/L)				
≤17.1	20	22		
>17.1	12	10	0.277	0.599
HbsAg				
Negative	23	27		
Positive	9	5	1.463	0.226
Chemotherapy regimens^b^				
GX	14	11		
X	18	21	0.591	0.442

^a^ Tumor location: cholangiocarcinoma (CCA) is best classified according to the primary, anatomic subtype as intrahepatic CCA (iCCA), perihilar CCA (pCCA), and distal CCA (dCCA).

^b^ Chemotherapy regimens: gemcitabine + capecitabine (GX); capecitabine monotherapy (X).

### 3.2 Survival analysis for AC + IM and AC groups

**FIGURE 2:**
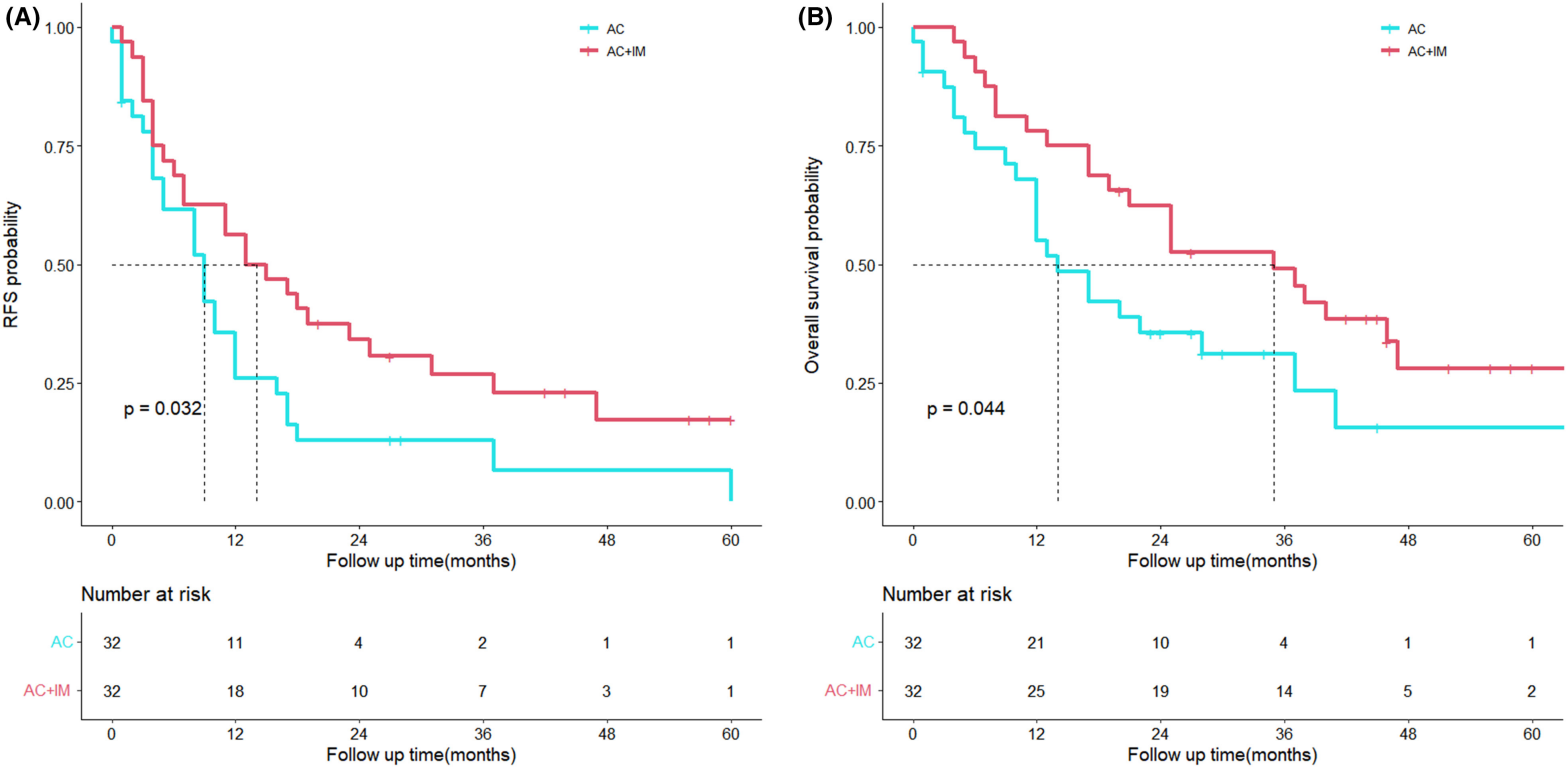
Kaplan–Meier analyses for RFS (A) and OS (B) based on postoperative immunotherapy. OS, overall survival; RFS, relapse‐free survival.

The AC + IM group demonstrated a significant improvement in RFS compared to the AC group (*p* = 0.032), as well as a significant enhancement in OS compared to the AC group (*p* = 0.044). The corresponding Kaplan–Meier survival curves illustrating these findings are depicted in Figure 2.

### 3.3 Prognostic factors for OS in the study cohorts after PSM analyzed by COX regression

Cox regression analysis unveiled that neural invasion (hazard ratio [HR], 2.20; 95% confidence interval [CI], 1.03–4.70; *p* = 0.041), positive lymph node metastasis (HR, 2.55; 95% CI, 1.32–4.94; *p* = 0.006), and immunotherapy (HR, 0.43; 95% CI, 0.23–0.80; *p* = 0.008) exerted significant impacts on OS across the groups, as illustrated in Table 3.

**TABLE 3:** Univariate and multivariate analysis of overall survival in the cohort after propensity score matching.

Characteristics	Univariate analyses			Multivariate analyses		
	HR	95% CI	*p* Value	HR	95% CI	*p* Value
Gender (male:female)	0.82	0.45–1.5	0.524			
Age (≤55:>55)	0.83	0.45–1.55	0.564			
Tumor location^a^						
iCCA	Ref					
pCCA	1.46	0.91–2.34	0.121			
dCCA	1.17	0.75–1.83	0.488			
Tumor differentiation						
High	Ref					
Moderate	1.19	0.62–2.26	0.600			
Low	0.69	0.38–1.26	0.228			
Microvascular invasion (absence:presence)	0.93	0.5–1.73	0.813			
Perineural invasion (absence:presence)	3.05	1.64–5.67	0	2.2	1.03–4.7	0.041
T stage (T1 or T2:T3 or T4)	1.46	1.05–2.03	0.025	1.12	0.76–1.66	0.569
Lymph node metastasis (absence:presence)	3.59	1.96–6.6	0	2.55	1.32–4.94	0.006
CEA (μmol/L) (≤5.00:>5.00)	1.7	0.91–3.15	0.094			
Preoperative CA199 (U/mL) (≤37.00:>37.00)	0.91	0.47–1.76	0.779			
Preoperative TBIL (μmol/L) (≤17.1:>17.1)	1.02	0.56–1.84	0.947			
HbsAg positive (absence:presence)	0.80	0.40–1.57	0.509			
Chemotherapy regimens (GX:X)^b^	0.95	0.60–1.52	0.843			
Immunotherapy (absence:presence)	0.49	0.27–0.88	0.018	0.43	0.23–0.8	0.008

^a^ Tumor location: cholangiocarcinoma (CCA) is best classified according to the primary, anatomic subtype as intrahepatic CCA (iCCA), perihilar CCA (pCCA), and distal CCA (dCCA).

^b^ Chemotherapy regimens: gemcitabine + capecitabine (GX); capecitabine monotherapy (X).

### 3.4 Survival analysis of patients with perineural invasion

**FIGURE 3:**
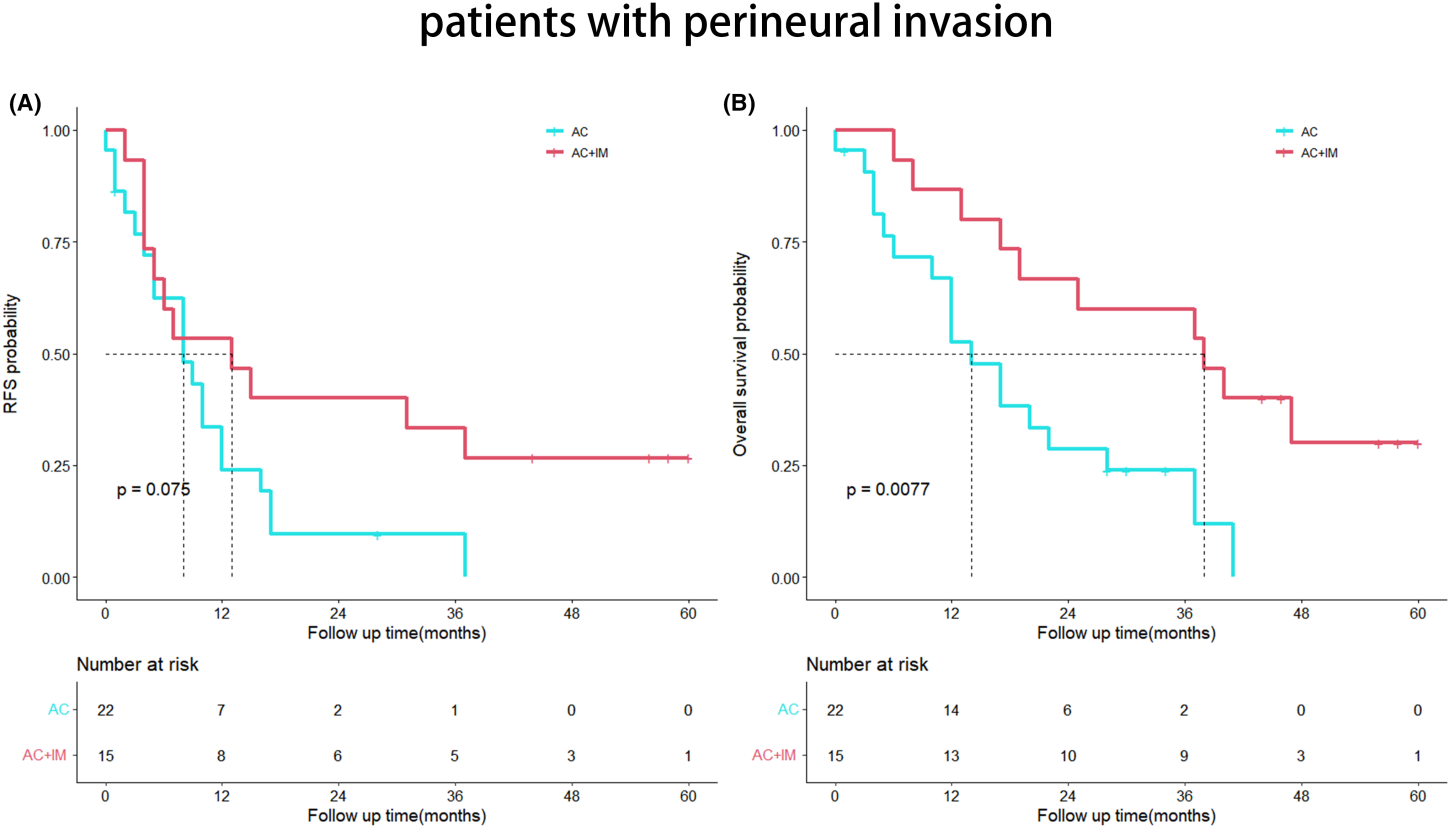
Kaplan–Meier analyses for RFS (A) and OS (B) based on postoperative immunotherapy. (A) RFS in the AC group versus the AC + IM group in patients with perineural invasion; (B) OS in the AC group versus the AC + IM group in patients with perineural invasion. AC, adjuvant chemotherapy; AC + IM, combined adjuvant chemotherapy and immunotherapy; OS, overall survival; RFS, relapse‐free survival.

The OS exhibited a significant improvement in the AC + IM group compared to the AC group (*p* = 0.0077). However, there was no significant difference observed in the RFS between the groups (*p* = 0.075). The Kaplan–Meier survival curves depicting these outcomes are presented in Figure 3.

### 3.5 Survival analysis of patients with lymph node metastasis

**FIGURE 4:**
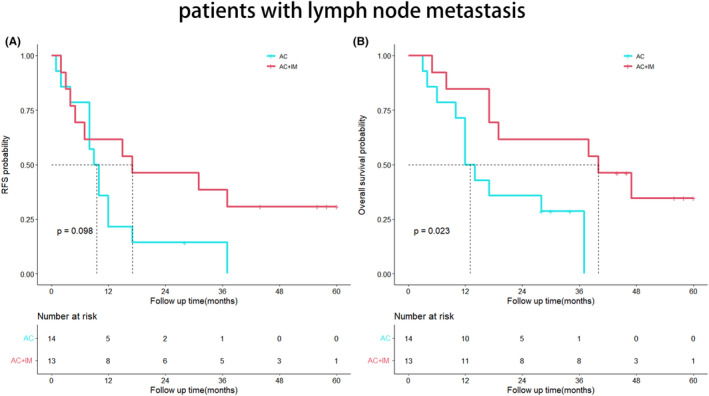
Kaplan–Meier analyses for RFS (A) and OS (B) based on postoperative immunotherapy. (A) RFS in the AC group versus the AC + IM group in patients with positive lymph node metastasis; (B) OS in the AC group versus the AC + IM group in patients with positive lymph node metastasis. AC, adjuvant chemotherapy; AC + IM, combined adjuvant chemotherapy and immunotherapy; OS, overall survival; RFS, relapse‐free survival.

No statistically significant difference was observed in RFS between the two groups (*p* = 0.098). However, the OS of the AC + IM group demonstrated a significant improvement compared to the AC group (*p* = 0.023), as depicted in Figure 4.

### 3.6 Analysis of adverse events in the AC + IM and AC groups

After PSM, a total of 64 patients were included for the analysis of adverse drug reactions. Among them, 51 cases (79.69%) experienced varying degrees of adverse reactions. In the AC + IM group, the adverse reactions exhibited the following hierarchy: foremost, nausea (63%) and diarrhea (50%), closely followed by a rash (50%). In the AC group, the adverse reactions displayed the following hierarchy: primarily, nausea (59%), followed by diarrhea (53%) and fatigue (47%). All patients experiencing Grade 1 and 2 adverse reactions exhibited improvement through observation or symptomatic treatment. In the AC + IM group, one patient encountered a severe systemic rash, which ameliorated following a week of hospitalization involving hormonal therapy and oral administration of loratadine tablets. The occurrence of Grade 3 and 4 adverse reactions was below 9% in both groups, and no fatalities resulted from adverse reactions. See Table 4 for more information.

**TABLE 4:** Postoperative adjuvant therapy alone or in combination with immunotherapy‐related adverse reactions after propensity score matching.

Adverse event	AC + IM groups (*n* = 32)		AC groups (*n* = 32)	
	Grade 1 and 2 (%)	Grade 3 and 4 (%)	Grade 1 and 2 (%)	Grade 3 and 4 (%)
Nausea	20 (63)	0	19 (59)	0
Diarrhea	16 (50)	0	17 (53)	0
Rash	16 (50)	1 (3)	13 (41)	2 (6)
Hand–foot syndrome	12 (38)	0	14 (44)	0
Fatigue	11 (34)	0	15 (47)	0
Leucopenia	11 (34)	2 (6)	12 (38)	2 (6)
Anemia	10 (31)	2 (6)	7 (22)	1 (3)
Thrombocytopenia	8 (25)	1 (3)	7 (22)	0
ALT elevation	5 (16)	0	7 (22)	0
AST elevation	4 (13)	0	6 (19)	0
TBIL elevation	4 (13)	0	5 (16)	0
Proteinuria	4 (13)	2 (6)	3 (9)	3 (9)
Hematuresis	3 (9)	2 (6)	3 (9)	2 (6)
Creatinine elevation	3 (9)	0	3 (9)	0

## 4 DISCUSSION

The efficacy of monotherapy with ICB in the treatment of cholangiocarcinoma remains inherently limited. The KEYNOTE‐158 study,^10^ a phase II clinical trial conducted on advanced cholangiocarcinoma patients who experienced progression after chemotherapy, evaluated the monotherapy of the PD‐1 inhibitor pembrolizumab. The objective response rate was found to be merely 5.8%. Fortunately, apart from monotherapy, the combination of immunotherapy with standard chemotherapy has been reported in multiple clinical trials to demonstrate favorable efficacy in advanced cholangiocarcinoma.^13^, ^14^, ^15^ However, the value of postoperative immunotherapy combined with chemotherapy in the cholangiocarcinoma remains uncertain.

We analyzed data from patients with cholangiocarcinoma who received either chemotherapy alone or a combination of chemotherapy and immunotherapy postoperatively. We observed that the combination of immunotherapy and chemotherapy demonstrated superior efficacy in surgically resected cholangiocarcinoma compared to chemotherapy alone. The AC + IM group exhibited significantly improved RFS and OS compared to the AC group (*p* = 0.032; *p* = 0.044). Furthermore, we identified immunotherapy as an independent factor influencing the OS of patients with cholangiocarcinoma after curative resection (*p* = 0.008).

The combination of immunotherapy and chemotherapy has yielded survival benefits in the treatment of advanced cholangiocarcinoma. A phase I trial^13^ conducted in advanced cholangiocarcinoma demonstrated the efficacy of ICB monotherapy compared to the combination of ICB and chemotherapy. The results revealed that the monotherapy group had a median progression‐free survival of 1.4 months and a median OS of 5.2 months, with only one patient (3%) achieving a PR. In contrast, the median OS for patients who received combination therapy was 15.4 months, with 11 patients (37%) achieving PR. Similarly, in another randomized trial,^15^ 128 patients with advanced cholangiocarcinoma underwent standard chemotherapy in combination with the PD‐L1 inhibitor, either with or without the CTLA4 inhibitor. Among the 47 patients in the chemotherapy + durvalumab group, 34 achieved objective responses (72%), while in the chemotherapy + durvalumab + CTLA4 inhibitor group of 47 patients, 33 achieved objective responses (70%). Moreover, the occurrence of Grade 3 and 4 adverse events was found to be below 53% in the entire study cohort, with no instances of adverse events resulting in fatality. A retrospective analysis by Ariizumi et al.^16^ on postoperative immunotherapy for cholangiocarcinoma revealed that the 5‐year OS rates of 36 patients receiving adjuvant chemotherapy and 34 patients receiving adjuvant immunotherapy were significantly higher than those of 57 patients undergoing liver resection alone (*p* = 0.0039). There was no significant difference between adjuvant chemotherapy and immunotherapy (*p* = 0.49), suggesting that immunotherapy holds certain value in the treatment of cholangiocarcinoma. Another retrospective analysis by Yu et al.^17^ suggested that the use of ICB after surgery in cholangiocarcinoma patients with mismatch repair deficiency could lead to survival benefits. An advantage of this study lies in its retrospective design, where the control group comprised 32 patients receiving adjuvant chemotherapy instead of undergoing surgery alone, potentially providing a better reflection of the efficacy of immunotherapy in postoperative cholangiocarcinoma. Furthermore, the study analyzed and compared the incidence of adverse reactions between chemotherapy combined with or without immunotherapy, indicating the favorable safety profile of the study's approach.

A previous study conducted at Shanghai Oriental Hepatobiliary Hospital in China, involving a cohort of 1031 patients, has underscored the significance of lymph node metastasis as a pivotal prognostic factor for postoperative cholangiocarcinoma.^18^ The expert consensus^19^ issued by the American Hepato‐pancreaticobiliary Association (AHPBA) indicates that 30%–35% of patients with intrahepatic cholangiocarcinoma exhibit lymph node metastasis. Kim et al.,^20^ utilizing PSM, revealed a significant extension in OS for the lymph node dissection group among 170 patients who underwent radical hepatectomy (*p* = 0.027). However, the association between lymph node metastasis and postoperative adjuvant therapy has been sparsely explored in the literature. In a multicenter study involving 1154 cholangiocarcinoma patients, Reames et al.^21^ demonstrated that those with stage N1 who received adjuvant chemotherapy experienced a notable enhancement in their 5‐year OS. Among patients with lymph node metastasis, the OS of the AC + IM group was superior to that of the AC group (*p* = 0.023). These findings indicate that cholangiocarcinoma patients with lymph node metastasis derive greater benefits from the combination of postoperative adjuvant chemotherapy and immunotherapy. Perineural invasion is frequently observed in the postoperative pathological examination of cholangiocarcinoma, imparting significant clinical implications. In a retrospective multicenter study,^22^ perineural invasion was detected in 239 (21.8%) out of 1095 patients, correlating with inferior RFS and OS. Likewise, Zhang et al.^23^ demonstrated that the perineural invasion‐negative group exhibited a more favorable prognosis in terms of OS (*p* < 0.0001). Another multicenter study^24^ revealed a perineural invasion incidence of 38% among cholangiocarcinoma patients. Notably, among those with perineural invasion, the AC + IM group displayed significantly improved OS compared to the AC group (40 months vs. 17 months, *p* = 0.0077). These findings reinforce the notion that patients with perineural invasion may derive greater benefits from postoperative adjuvant therapy, aligning with previous investigations.^23^, ^24^, ^25^

Our study possesses certain limitations. Firstly, it is essential to note that this study represents a nonrandomized, retrospective analysis conducted on a relatively limited patient cohort. Secondly, the majority of patients were predominantly from southern region of China, which may pose a potential limitation to the generalizability of our findings.

## 5 CONCLUSION

In summary, the combination of adjuvant chemotherapy and immunotherapy demonstrates enhanced RFS and OS rates among patients who have undergone surgical resection for cholangiocarcinoma. Notably, perineural invasion, lymph node metastasis, and immunotherapy emerge as independent prognostic factors for postoperative cholangiocarcinoma patients. Furthermore, the administration of adjuvant chemotherapy alongside immunotherapy proves to be a safe therapeutic approach for this patient population.

## AUTHOR CONTRIBUTIONS

**Xiao‐hui Li:** Conceptualization (equal); writing – original draft (equal). **En‐liang Zhou:** Data curation (equal). **Chong‐yu Zhao:** Conceptualization (equal). **Bo‐Kang Cui:** Supervision (equal). **Xiao‐yuan Dong:** Validation (equal). **Hang Du:** Writing – review and editing (equal). **Xiao‐jun Lin:** Conceptualization (equal); resources (equal).

## FUNDING INFORMATION

No external funding support was received for this study.

## CONFLICT OF INTEREST STATEMENT

We affirm that there are no conflicts of interest among all coauthors involved in this study.

## ETHICS STATEMENT

This study was conducted in accordance with the ethical standards set forth by the institutional review board of Sun Yat‐sen University Cancer Center, with approval granted under the reference number B2022‐492‐01. All procedures involving human participants adhered to the principles outlined in the 1964 Helsinki Declaration and its subsequent amendments.

## Data Availability

The article presents the data utilized in this study.
